# Chronic radiation exposure at Chernobyl shows no effect on genetic diversity in the freshwater crustacean, *Asellus aquaticus* thirty years on

**DOI:** 10.1002/ece3.5478

**Published:** 2019-08-24

**Authors:** Neil Fuller, Alex T. Ford, Adélaïde Lerebours, Dmitri I. Gudkov, Liubov L. Nagorskaya, Jim T. Smith

**Affiliations:** ^1^ Institute of Marine Sciences, School of Biological Sciences University of Portsmouth Portsmouth UK; ^2^ Department of Freshwater Radioecology Institute of Hydrobiology Kiev Ukraine; ^3^ Applied Science Center for Bioresources of the National Academy of Sciences of Belarus Minsk Belarus; ^4^ School of Earth & Environmental Sciences University of Portsmouth Portsmouth UK

**Keywords:** Chernobyl, crustacean, genetic diversity, genotyping‐by‐sequencing

## Abstract

Analysis of genetic diversity represents a fundamental component of ecological risk assessments in contaminated environments. Many studies have assessed the genetic implications of chronic radiation exposure at Chernobyl, generally recording an elevated genetic diversity and mutation rate in rodents, plants, and birds inhabiting contaminated areas. Only limited studies have considered genetic diversity in aquatic biota at Chernobyl, despite the large number of freshwater systems where elevated dose rates will persist for many years. Consequently, the present study aimed to assess the effects of chronic radiation exposure on genetic diversity in the freshwater crustacean, *Asellus aquaticus*, using a genome‐wide SNP approach (Genotyping‐by‐sequencing). It was hypothesized that genetic diversity in *A. aquaticus* would be positively correlated with dose rate. *A. aquaticus* was collected from six lakes in Belarus and the Ukraine ranging in dose rate from 0.064 to 27.1 µGy/hr. Genotyping‐by‐sequencing analysis was performed on 74 individuals. A significant relationship between geographical distance and genetic differentiation confirmed the Isolation‐by‐Distance model. Conversely, no significant relationship between dose rate and genetic differentiation suggested no effect of the contamination gradient on genetic differentiation between populations. No significant relationship between five measures of genetic diversity and dose rate was recorded, suggesting that radiation exposure has not significantly influenced genetic diversity in *A. aquaticus* at Chernobyl. This is the first study to adopt a genome‐wide SNP approach to assess the impacts of environmental radiation exposure on biota. These findings are fundamental to understanding the long‐term success of aquatic populations in contaminated environments at Chernobyl and Fukushima.

## Graphical abstract

This study utilized a genotyping by sequencing method to provide a novel assessment of the impacts of chronic radiation exposure at Chernobyl on a freshwater crustacean. No significant impact of over 30 years of radiation exposure on genetic variation was recorded. These findings are fundamental to understanding the long‐term success of aquatic populations in contaminated environments at Chernobyl and Fukushima.

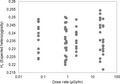

## 1 INTRODUCTION

Studies of the impacts of contaminants on genetic diversity have increased in recent years owing to the advent of affordable DNA sequencing services and the recognized importance of maintaining genetic diversity in the conservation of wildlife populations (Frankham, Bradshaw, & Brook, 2014; Giska, Babik, Gestel, Straalen, & Laskowski, 2015; Rumisha et al., 2017). Alterations to genetic diversity may lead to reduced susceptibility to environmental change (Ehlers, Worm, & Reusch, 2008) and increased extinction potential. Furthermore, changes to genetic diversity may reflect long‐term pollutant impacts more adequately than many commonly used biomarkers which reflect transient stress on populations (Anderson et al., 1994; Bickham, Sandhu, Hebert, Chikhi, & Athwal, 2000). Ionizing radiation is a known mutagen with the capacity to cause a range of alterations to the genome, including chromosomal aberrations, micronuclei formation, and gene mutations (Morgan, 2003). While the genetic consequences of acute, high doses of radiation are well understood (Little, Nagasawa, Pfenning, & Vetrovs, 1997; Tucker, Cofield, Matsumoto, Ramsey, & Freeman, 2005), the impacts of chronic sublethal doses over multiple generations are comparatively less clear (Baker et al., 2017).

Following the 1986 Chernobyl accident, the worst nuclear accident in history, a number of studies assessed genetic effects of radiation exposure on both humans and wildlife (Dubrova et al., 1996, 1997; Ellegren, Lindgren, Primmer, & Møller, 1997; Matson, Rodgers, Chesser, & Baker, 2000). Elevated mutation rates have been recorded in the offspring of liquidators involved in the clean‐up operation (Weinberg et al., 2001) and among children born in heavily contaminated areas (Dubrova et al., 1996, 1997). Similarly, studies have demonstrated elevated genetic diversity and mutation rates in a range of wildlife inhabiting contaminated areas (Baker et al., 2017; Ellegren et al., 1997). However, these findings are not ubiquitous, with a number of studies finding no significant effects of Chernobyl‐derived radiation on genetic variation (DeWoody, 1999; Furitsu et al., 2005; Livshits et al., 2001).

Though the majority of studies suggest an increase in genetic diversity and mutation rates in contaminated areas of Chernobyl, a reduction in genetic diversity could also have occurred owing to population bottlenecks induced by high dose rates immediately following the accident. Bottleneck events occur when the size of a population is reduced due to contaminant exposure, leading to a small subset of genotypes available for recovery and expansion (van Straalen & Timmermans, 2002). This would lead to reduced genetic diversity due to the direct removal of genotypes, known as “genetic erosion.” To the author's knowledge, no study has demonstrated a reduced genetic diversity in wildlife at Chernobyl.

Despite the large number of studies assessing genetic effects of environmental radiation on natural populations of rodents (Baker et al., 2017; Matson et al., 2000; Wickliffe et al., 2003) and plants (Geras'kin & Volkova, 2014; Kovalchuk, Dubrova, Arkhipov, Hohn, & Kovalchuk, 2000; Tsyusko, Smith, Oleksyk, Goryanaya, & Glenn, 2006; Volkova et al., 2018), only two studies have considered effects on aquatic biota. Theodorakis and Shugart (1998) studied genetic variation using random amplified polymorphic DNA (RAPD) methods in mosquito fish (*Gambusia affinis*) inhabiting ponds contaminated with radioactive waste from the US Department of Energy's Oak Ridge facility. The authors recorded increased genetic diversity in *G. affinis* individuals inhabiting two contaminated ponds (dose rates of 50 and 1,313 µGy/hr) as opposed to individuals from two reference populations. However, one of the aforementioned sites is also heavily contaminated with a suite of other genotoxicants (e.g., heavy metals and polycyclic aromatic hydrocarbons, Theodorakis & Shugart, 1998), meaning any observed effects cannot be attributed solely to radiation. The Chernobyl area is heterogeneously contaminated by a range of radionuclides, including ^90^Sr, ^137^Cs and transuranium isotopes (e.g., ^238^Pu, ^239,240^Pu, and ^241^Am, Beresford et al., 2018). Doses to aquatic macroinvertebrates are dominated by external γ and β radiation from ^137^Cs and ^90^Sr (Murphy, Nagorskaya, & Smith, 2011).

To the authors' knowledge, only a single study has considered radiation effects on genetic diversity in aquatic biota at Chernobyl, despite the large number of closed lake systems in the area where biota are chronically exposed to low‐dose radiation. Fetisov, Rubanovich, Slipchenko, and Shevchenko (1992) studied genetic and morphological differences in seven populations of the zebra mussel, *Dreissena polymorpha,* in the Chernobyl area using an allozyme method. Based on data for five loci, no significant influence of radiation on *D. polymorpha* genetic structure was recorded, though thermal regime was found to influence genetic diversity. However, the use of allozymes in genetic diversity studies has been questioned, particularly at small spatial scales and using a small number of loci (Turlure, Vandewoestijne, & Baguette, 2014). Furthermore, the influence of radiation was not the direct focus of the study and no dose rates were provided, necessitating further robust study into the impacts of chronic radiation exposure on the genetic diversity of aquatic invertebrates. The 2011 accident at the Fukushima Dai‐Ichi nuclear power plant led to further contamination of marine and freshwater environments that will persist for decades (IAEA, 2015). This further emphasizes the need for robust studies of the long‐term genetic consequences of environmental radiation on aquatic biota.

Many of the studies of radiation‐induced genetic changes highlighted above used techniques such as restriction fragment length polymorphisms (RFLPs, e.g., Theodorakis & Shugart, 1998), amplified fragment length polymorphisms (AFLPs, e.g., Volkova et al., 2018), or microsatellites (Ellegren et al., 1997). Recent studies have shown that genome‐wide analysis of single‐nucleotide polymorphisms (SNPs) provides less biased measures of genetic diversity as compared to these more traditional techniques (Fischer et al., 2017). SNPs are more widely abundant across the genome as compared to microsatellites and RFLP‐based techniques and are generally more reproducible (Davey et al., 2011; Schlötterer, 2004). The advent of affordable sequencing coupled with continual advancements in technologies have led to the application of genome‐wide SNP approaches to a wide range of questions, including evolutionary history (Pollinger et al., 2010), conservation (Larson et al., 2014), and ecotoxicology (Giska et al., 2015).

Taking into account these knowledge gaps, the present study aimed to assess the impact of chronic radiation exposure on the freshwater crustacean, *Asellus aquaticus*, along a gradient of contamination at Chernobyl using a genome‐wide SNP approach. Genotyping‐by‐sequencing (GBS) is a simple, cost‐effective reduced representation method for assessing a vast number of SNPs across the genome (Narum, Buerkle, Davey, Miller, & Hohenlohe, 2013). GBS has been applied to a wide range of both model and nonmodel organisms and has been demonstrated to be a useful technique for population genomic analyses even where reference genomes are not available (White, Perkins, Heckel, & Searle, 2013). *Asellus aquaticus* is a detrivorous isopod crustacean commonly found in freshwater systems across Europe. Crustaceans are one of the International Commission on Radiological Protection's (ICRP) eight reference animals and plants (RAPs), meaning these organisms will be used to support the evolving system for environmental radioprotection (ICRP, 2007). *A. aquaticus* is commonly used in ecotoxicology studies of sediment‐borne contaminants (De Lange, Haas, Maas, & Peeters, 2005; McCahon & Pascoe, 1988) and has been used as an indicator of water quality (Whitehurst, 1991). Previous studies have demonstrated no effects of chronic radiation exposure at Chernobyl on the development and reproduction of *A. aquaticus* (Fuller, Ford, Nagorskaya, Gudkov, & Smith, 2018; Fuller, Smith, Nagorskaya, Gudkov, & Ford, 2017). However, to the authors knowledge no studies have been conducted on the lethal tolerance of *A. aquaticus* to ionizing radiation.

Based on the number of studies documenting an increase in genetic diversity in biota at Chernobyl (Baker et al., 2017; Matson et al., 2000; Volkova et al., 2018), we hypothesized that populations of *A. aquaticus* would display elevated genetic diversity along a gradient of radionuclide contamination at Chernobyl. In addition, the influence of the gradient in dose rate on genetic differentiation (measured as *F*_st_) was assessed following Giska et al., (2015) and Rumisha et al., (2017).

## 2 MATERIALS AND METHODS

### 2.1 Field sampling and collection of *A. aquaticus*

**Figure 1:**
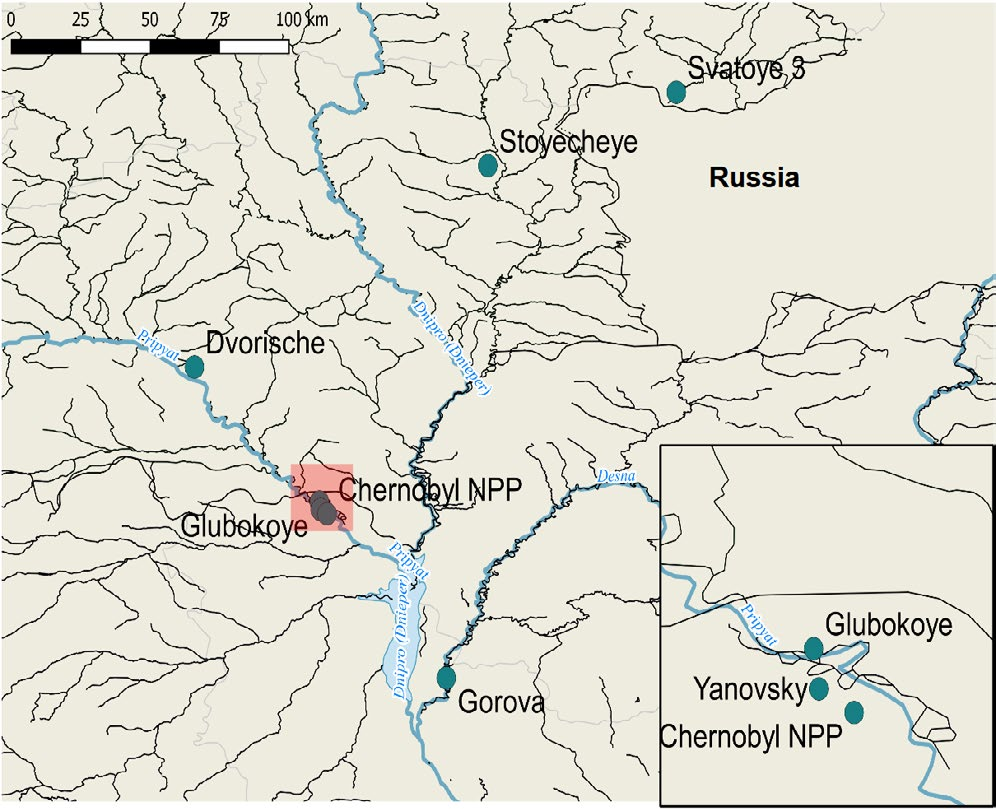
Map of the six study sites sampled for *Asellus aquaticus* in relation to distance from the Chernobyl Nuclear Power Plant (CNPP). Map generated using qGIS (v2.18) with data from DIVA‐GIS (Hijmans, Guarino, Cruz, & Rojas, 2001)

Field sampling was conducted in May‐June of 2016 at six lakes in Belarus and Ukraine (see Figure 1) as described in Fuller et al., (2017), Fuller et al., (2018). Sampling sites were selected based on historical measurements of radioactivity and exposure to a gradient of radionuclide contamination. *A. aquaticus* were collected by kick netting in littoral zones using a 1 mm mesh size net (EFE), sorted lakeside and immediately preserved in 96% Ethanol. A total of 455 *A. aquaticus* individuals were collected across all sites during the sampling period. Numbers of *A. aquaticus* individuals sequenced are shown in Section 2.4.

### 2.2 Environmental conditions

A range of different environmental parameters were measured at three subsites of each lake using a multiparameter probe (HANNA Instruments 9828). Sampling dates and environmental conditions are shown in Table 1.

**Table 1:** Environmental conditions, location, sampling date, and total dose rate at six sites of varying contamination in Belarus and the Ukraine

Site	Sampling date	Latitude	Longitude	Dose rate (µGy/hr)	Temperature (°C)	Oxygen saturation (%)	pH	Conductivity (µs/cm)
Gorova	11/6/2016	50.70	30.70	0.064	22.4 ± 0.05	113 ± 16.2	8.60 ± 0.02	256 ± 0.41
Dvorische	29/05/2016	52.01	29.43	0.691	23.2 ± 0.06	80 ± 1.17	7.60 ± 0.17	197 ± 0.15
Stoyecheye	27/05/2016	52.86	30.91	0.774	22 ± 0.05	102 ± 2.00	8.30 ± 0.02	241 ± 1.48
Svatoye	24/05/2016	53.17	31.86	2.03	20.1 ± 0.23	92 ± 1.80	8.00 ± 0.15	114 ± 0.70
Yanovsky Crawl	05/06/2016	51.41	30.07	20.42	20.2 ± 0.11	140 ± 2.90	9.00 ± 0.04	265 ± 0.97
Glubokoye	03/06/2016	51.44	30.06	26.4	23.6 ± 0.06	112 ± 14.10	7.60 ± 0.18	199 ± 1.22

Note

Error bars are standard deviations based on measurements at three different subsites of each lake.

### 2.3 Calculation of dose rates

A full description of the methods used to calculate doses to *A. aquaticus* is presented in Fuller et al., (2017). Briefly, external and internal dose rates to *A. aquaticus* were calculated based on decay‐corrected deposition values for radiocaesium and strontium (in 1986) and average activity concentrations in water at various depths measured in 2003. Accurate dose conversion coefficients (DCCs) for *A. aquaticus* were generated within the ERICA tool (Version 1.2) by user‐inputted geometry (height = 2.2 mm, width = 1.7 mm, length = 4.7 mm, and mass = 4.1 mg) and used for subsequent dose assessments using ERICA. Total dose rates for *A. aquaticus* at each of the six lakes are shown in Table 1.

### 2.4 Genomic DNA extractions and quality control

DNA was extracted from a total of 74 *A. aquaticus* individuals using a QIAGEN DNeasy Blood & Tissue Kit (QIAGEN) following some amendments to the manufacturer protocol to maximize DNA yield. Individuals were first homogenized in 180 µl of Buffer ATL. Following addition of 40 µl Proteinase K, samples were incubated at 56°C for 16 hr to allow for lysis to occur. After lysis, samples were centrifuged at 20,000 *g* for 5 min and the supernatant transferred to a new microcentrifuge tube. This step was performed to remove the nonlysed, largely chitinous tissue as this may impact the quality of DNA for downstream applications in *A. aquaticus* (Verovnik, Sket, Prevorčnik, & Trontelj, 2003). Samples were then processed according to the animal tissue spin‐column protocol outlined in the QIAGEN Blood and Tissue Kit. Quality of extracted genomic DNA was assessed using a NanoDrop spectrophotometer, and restriction enzyme digests with Hind*III* (Thermo Fisher) were performed on 10% of DNA samples to assess suitability for genomic library construction. Digests were performed according to manufacturer protocol and visualized on a 1% agarose gel.

### 2.5 Genotyping‐by‐sequencing

A full description of GBS methods is provided in Elshire et al., (2011). Briefly, extracted DNA was arrayed on to a 96‐well sealed fully skirted PCR plate (Applied BiosystemsUS) and shipped to the Cornell University Genomic Diversity Facility for GBS analysis. Following optimization, Pstl (CTGCAG) was selected as the most appropriate restriction enzyme owing to the greater number of fragment sizes generated. Each DNA sample was digested with Pstl and ligated with a barcoded and a common adaptor. Following ligation of the adaptor, samples were pooled within a single Eppendorf tube and purified using a Qiagen QIAquick PCR Purification Kit (Qiagen). The library was then subjected to the polymerase chain reaction (PCR) using primers that corresponded to the ligated adaptors. After appropriate dilution of samples, sequencing was performed using the Illumina Hi‐Seq 2000 at the Cornell University Core Laboratories Centre.

### 2.6 Bioinformatics and SNP calling

Bioinformatics analysis was conducted by collaborators at the Genomic Diversity Facility using the UNEAK (Universal Network‐Enabled Analysis Kit, Lu et al., 2013) pipeline and the TASSEL software version v 3.0 (Trait Analysis by aSSociation, Evolution and Linkage, Bradbury et al., 2007). The UNEAK pipeline was developed to overcome the issues associated with SNP discovery in absence of a reference genome and has been shown to provide accurate (>92% accuracy) de novo genotype calling (Lu et al., 2013; Torkamaneh, Laroche, & Belzile, 2016). The UNEAK pipeline works by first trimming all sequence reads to 64 bp. Reads of 64 bp that are identical are then identified as tags, and pairwise alignment identifies pairs of tags with a single base pair mismatch. Such tags are aligned to form a network and these networks are pruned to remove putative sequencing errors (low‐frequency alleles). Pruning was conducted according to an error rate threshold parameter of 0.01%. Only reciprocal tag pairs are used for subsequent SNP calling, meaning only biallelic loci were studied. VCF tools (v0.1.12a) were then used to calculate depth and missingness statistics for generated SNP files. Heterozygosity and minor allele frequencies were also calculated using VCF tools.

### 2.7 Statistical analyses

The R package SNPrelate (Zheng et al., 2012) was used to calculate additional diversity statistics and to create a principal component analysis (PCA) plot of genotypes from filtered SNP data. A matrix of *F*_st_ values were calculated according to the method of Weir and Cockerham (1984). Other measures of genetic diversity such as Tajima's *D*, nucleotide diversity, and expected heterozygosity were calculated using the R Package PopGenome (Pfeifer, Wittelsbürger, Ramos‐Onsins, & Lercher, 2014). In order to test for the presence of Isolation‐by‐distance (IBD), a Mantel test was used with geographical distance (in km) and *F*_st_ values via the R package vegan (Oksanen et al., 2013). To test for the influence of the gradient in dose rate on genetic differentiation, a partial mantel test was used based on Pearson's product‐moment correlation with 1,000 permutations. This test measures the association between two matrices (e.g., genetic distance and Euclidean distance in dose rate) while accounting for a third potentially confounding matrix (geographical distance). The association between calculated genetic diversity measures and dose rates was calculated using Spearman's rank order correlation coefficient. Power analysis was employed to determine the probability of seeing different effect sizes based on sample size using the R package “pwr” (Champely et al., 2018).

## 3 RESULTS

### 3.1 Data quality and coverage

Illumina sequencing of 74 individuals on one lane yielded a total of 492 402 538 reads. Of these, 372,029,165 were classed as “good” reads containing a unique barcode. Depth of coverage was on average 25.74. A depth of coverage >20 times is preferred in SNP studies to reduce the uncertainty associated with calling SNPs following low‐coverage sequencing (e.g., 5 × depth of coverage, Nielsen, Paul, Albrechtsen, & Song, 2011). The UNEAK pipeline identified a total of 32,321 SNPs which was trimmed to 14,463 following the data filtration steps.

### 3.2 Genetic differentiation and isolation‐by‐distance

**Figure 2:**
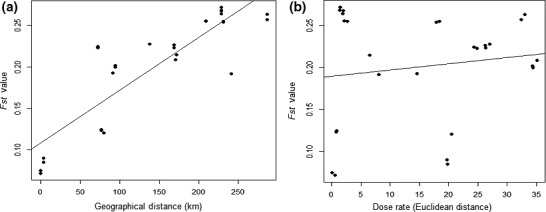
Relationship between (a) geographical distance and genetic differentiation (*F*_st_) and (b) gradient in dose rate and genetic differentiation in *Asellus aquaticus* from six lakes of varying contamination in the Chernobyl area

Genetic differentiation values (*F*_st_) are shown in Table 2. Greatest values were observed between Svatoye and Glubokoye lakes (0.272), suggesting these populations were the most genetically isolated. *F*_st_ values >0.15 are generally considered to indicate significant differentiation (Frankham, Briscoe, & Ballou, 2002). The smallest *F*_st_ values were recorded between Glubokoye and Yanovsky (0.085), the two closest sites geographically. A highly significant relationship between geographical distance in kilometers and genetic differentiation was recorded (see Figure 2a, Mantel Test, *r* = .786, *p* < .01), confirming the Isolation‐by‐distance hypothesis.

**Table 2:** Pairwise genetic differentiation (*F*_st_) values in *Asellus aquaticus* collected from six lakes of varying contamination in Belarus and the Ukraine

	Dvorische	Glubokoye	Svatoye Lake	Yanovsky Crawl	Stoyecheye	Gorova
Dvorische						
Glubokoye	0.1235103					
Svatoye Lake	0.255504	0.2719511				
Yanovsky Crawl	0.1206123	0.08523209	0.254953			
Stoyecheye	0.2278073	0.2233065	0.223041	0.21475		
Gorova	0.2086033	0.1999745	0.263433	0.1927364	0.1918563	

**Figure 3:**
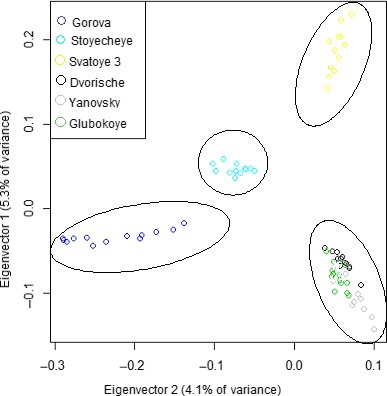
Principal component analysis of genotypes based on genome‐wide SNP data of *A. aquaticus* collected from six lakes along a gradient of radionuclide contamination at Chernobyl

Conversely, no significant effect of the gradient in dose rate (Euclidean distance) on genetic differentiation was recorded when accounting for geographical distance (see Figure 2b, Partial Mantel test, *r* = .251, *p* > .05). This indicates that dose rate did not significantly influence genetic differentiation among populations at Chernobyl. However, geographical distance was found to be a driver of genetic differentiation. A principal component analysis (PCA) plot of genotypes based on genome‐wide filtered SNP data is shown in Figure 3. Two of the highly contaminated sites, Glubokoye and Yanovsky Crawl (dose rates of 26.4 and 20.4 µGy/hr respectively) along with a site of low‐level contamination (Dvorische, dose rate of 0.691 µGy/hr) were differentiated from other sites in terms of genetic similarity. These sites were located in closest proximity geographically, reinforcing the strong influence of the geographical gradient on determining genetic similarity. Furthermore, all of the previously mentioned lakes (Dvorische, Yanovsky Crawl and Glubokoye) are floodplain lakes that historically would have been linked to the Pripyat river, reinforcing the potential for gene flow and therefore genetic similarity of these populations.

### 3.3 Genetic diversity

**Figure 4:**
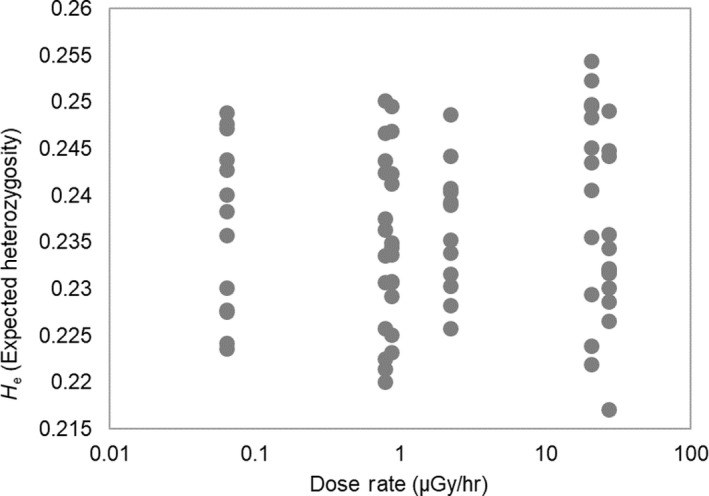
Relationship (Spearman's *ρ *= −.110, *p* > .05) between *H*_e_, expected heterozygosity, in *Asellus aquaticus* and dose rate at six sites of varying radionuclide contamination in Belarus and the Ukraine

Calculated genetic diversity statistics for each population are shown in Table 3. Expected heterozygosity (*H*_e_), the most commonly used measure of gene diversity (Nei, 1973), ranged from 0.234 at Glubokoye to 0.241 at Yanovsky Crawl. No significant relationship between *H*_e_ and total dose rate was recorded (see Figure 4, *n* = 74, Spearman's *ρ* = −.110, *p* > .05). Nucleotide diversity (*π*), a measure of polymorphism within the population, ranged from 0.0016 at Svatoye to 0.0026 at Gorova. No significant relationship between nucleotide diversity and dose rate was recorded (Spearman's *ρ* = −.333, *p* > .05).

**Table 3:** Calculated genetic diversity measures based on genome‐wide single‐nucleotide polymorphism data in *Asellus aquaticus* from six lakes in the Chernobyl region

	*N*	*H*_e_	*H*_0_	*F*	*π*	Tajima's *D*
Dvorische	12	0.23426 ± 0.010	0.12343 ± 0.034	0.47537 ± 0.134	0.001779881	−1.074
Glubokoye	13	0.23373 ± 0.008	0.10739 ± 0.020	0.54170 ± 0.079	0.002438229	−1.099
Gorova	13	0.23677 ± 0.009	0.13059 ± 0.031	0.44943 ± 0.126	0.002669943	−0.659
Stoyecheye	12	0.23521 ± 0.008	0.12750 ± 0.029	0.45792 ± 0.114	0.002215685	−0.671
Svatoye	12	0.23646 ± 0.007	0.14392 ± 0.050	0.39342 ± 0.186	0.001649979	−0.612
Yanovsky Crawl	12	0.24123 ± 0.011	0.14549 ± 0.029	0.40218 ± 0.145	0.002023974	−0.984

Notes

Values are shown as ± *SD* for genetic diversity measures calculated at the individual level (e.g., *H*_e_, *H*_0_ and *F*).

*H*_e_ = expected heterozygosity, *H*_0_ = observed heterozygosity, *F* = inbreeding coefficient, and *π* = nucleotide diversity.

Similarly, no significant relationship between dose rate and observed heterozygosity (*H*_o_) or the within population inbreeding coefficient, *F*, was recorded (Spearman's *ρ* = −.018, *p* > .05 and *ρ* = −.031, *p* > .05, respectively). Coupled with the lack of a relationship between genetic differentiation and dose rate, this provides evidence that genetic patterns in *A. aquaticus* at Chernobyl were not influenced by radiation dose rate. Tajima's *D* statistic was <0 for all populations (see Table 3), with the lowest value recorded at Glubokoye (−1.099) and the greatest value recorded at Svatoye (−0.612). A moderate negative relationship between Tajima's *D* values and dose rate was recorded, though this was nonsignificant (Spearman's *ρ* = −.5, *p* > .05).

## 4 DISCUSSION

The present study hypothesized that genome‐wide estimates of genetic diversity in the freshwater crustacean *Asellus aquaticus* would be positively related to the total dose rate at six lakes in the Chernobyl region. No significant relationship between five different genetic diversity metrics and dose rate was recorded, leading to rejection of this hypothesis. A strong significant effect of geographical distance on genetic differentiation was evidenced, suggesting Isolation‐by‐distance. Conversely, the gradient in radionuclide contamination was not found to impact genetic differentiation. Calculated genetic diversity measures for *A. aquaticus* in the present study were within the range of other areas of Europe as recorded by Verovnik, Sket, and Trontelj (2005) and Konec, Prevorčnik, Sarbu, Verovnik, and Trontelj (2015). Verovnik et al., (2005) recorded mean values for nucleotide diversity, *π*, in populations of *A. aquaticus* across central and Eastern Europe of 0.00189 ± 3.79E‐06 using mitochondrial DNA methods. Values obtained within the present study were broadly similar, ranging from 0.00165 to 0.00267.

The suggestion of IBD in the present study is in disagreement with previous work conducted by Verovnik et al., (2005) who studied the mitochondrial DNA and 28s rDNA sequences of *A. aquaticus* at a continental scale and found no evidence of IBD. The present study focused largely on closed lake systems with limited connectivity (though Dvorische, Yanovsky Crawl, and Glubokoye are floodplain lakes of the Pripyat river which would have been connected historically), which is fundamental to structuring *A. aquaticus* populations given known limitations on dispersal capacity (Sworobowicz et al., 2015; Verovnik et al., 2005). This may explain the presence of IBD in the present study, as it is assumed limited gene flow would have occurred between enclosed, isolated populations. Methodological differences in the calculation of genetic differentiation and techniques used to assess genetic structure may further explain the differences between studies. For example, studies have shown significant variation in the ability of genetic markers, for example, microsatellite or SNP based techniques to distinguish genetic differentiation of populations (Bradbury et al., 2015).

Gene flow between populations is often thought to mask the effects of pollutants on genetic patterns, even in species with known limited dispersal capacity (Giska et al., 2015; Theodorakis, Bickham, Lamb, Medica, & Lyne, 2001). However, the moderate to high levels of genetic differentiation observed between populations in the present study (*F*_st_ values up to 0.27) and the confirmation of IBD suggests that gene flow and homogenization of populations is likely not responsible for the lack of a relationship between genetic variables and dose rate. This suggests that current dose rates received by *A. aquaticus* at Chernobyl (maximum of 27.1 µGy/hr) are either insufficient to cause a high rate of mutations and subsequent elevated genetic diversity or that effects may have occurred previously but populations have recovered. These findings contradict the majority of studies which record an increase in genetic diversity in a range of nonhuman organisms at Chernobyl (Baker et al., 2017; Ellegren et al., 1997).

One possible explanation for these differences may be that *A. aquaticus* populations have not been historically exposed to dose rates as high as those for rodents and pines that have been shown to have altered genetic diversity (e.g., Baker et al., 2017; Volkova et al., 2018). For example, many of the studies of genetic diversity in bank voles at Chernobyl have monitored populations from the Red Forest (Baker et al., 2017; Matson et al., 2000). The Red Forest refers to the highly contaminated area adjacent to the CNPP where acute doses led to mortality in a 4–6 km^2^ zone of pines immediately following the accident (Kryshev, Sazykina, & Beresford, 2005). Dose rates in this area remain extremely high to date, with maximum air dose rates of 200 µGy/hr recorded in some “hotspots” within the forest (N.A. Beresford, pers. comm). Studies on the accumulation of radionuclides in small mammals within this area in the late 1990s suggested that chronic dose rates could be as high as 86 mGy/day for the duration of their lifecycle (Chesser et al., 2000, 2001). Similarly, many of the studies demonstrating genetic effects in scots pine are based on trees receiving doses orders of magnitude higher than the present study (Kuchma, Vornam, & Finkeldey, 2011; Vornam, Arkhipov, & Finkeldey, 2012), though effects at lower doses have also been recorded (Geras'kin & Volkova, 2014; Volkova, Geras'kin, & Kazakova, 2017).

Maximum external dose rates in aquatic systems immediately following the accident were 100–200 mGy/day from bottom sediments (Kryshev et al., 2005), showing a relatively rapid decline to 20–50 mGy/day within two months owing to the decay of short‐lived radionuclides (Kryshev et al., 2005). Dose rates further declined through 1986, with mean dose estimates for benthic fish in 1986 being 22 ± 9.0 mGy/day, significantly lower than the previously mentioned Red Forest area (Kryshev & Sazykina, 2012). Though these dose rates were calculated for the Chernobyl cooling pond, doses at the most contaminated site in the present study, Glubokoye, were likely similar. While these dose rates would be expected to cause significant sublethal effects, it is unlikely that direct mortality and a resultant bottleneck was induced in crustaceans based on existing laboratory sensitivity data (see Fuller, Lerebours, Smith, & Ford, 2015 for review). In crabs, for example, chronic exposure to ^60^Co dose rates of 6,960 mGy/day were necessary to elicit mortality in *Callinectes sapidus*, over an order of magnitude greater than doses in the immediate aftermath of the accident (Engel, 1967).

Taking this into account, it is unlikely that the Chernobyl accident would have caused a significant bottleneck in crustacean populations, accounting for the lack of a reduced genetic diversity observed in the present study. This was further reinforced by Murphy et al., (2011), who recorded no significant reduction in the abundance of macroinvertebrates in eight Chernobyl‐affected lakes in 2003 and 2004. However, it is important to consider that the lack of observed genetic effects in the present study does not indicate that discernible effects have not occurred in *A. aquaticus* as a consequence of the Chernobyl accident. As proposed by DeWoody, (1999) population sampling may have taken place at time wherein radiation‐induced mutation and elevated genetic diversity may have facilitated a more rapid recovery from a previous bottleneck and the resultant reduced genetic diversity.

One limitation of this study was the limited number of individuals sequenced per site and lack of an adequate number of samples collected at various time points (see Matson et al., 2000 for discussion). For analysis of the relationship between total dose rate and a range of gene diversity measures, 74 samples were analyzed. For an 80% chance of detecting a “medium” effect size of *r* = .3 (Cohen, 1977), 84 samples would be necessary assuming a significance level of 0.05. A much larger sample size of >700 samples would be necessary for the same conditions for a “small” effect size of 0.1 (Cohen, 1977). It is therefore possible that radiation may have induced a minor effect on genetic diversity in *A. aquaticus* that the present study may not have had the necessary statistical power to detect. Furthermore, effective population size was not estimated in this study. Under neutral theory, a given population's genetic diversity is dependent on both the effective population size and the mutation rate (Kimura, 1983). Larger effective populations typically show greater genetic diversity than smaller populations (Hague & Routman, 2016), though some comparative studies have questioned this relationship (Bazin, Glémin, & Galtier, 2006). The conclusions of the present study are therefore based on the assumption of similar effective population sizes at these locations. In order to discriminate the drivers regulating genetic variation at the population level, multiple sampling years of a greater number of individuals and a robust understanding of demographic and ecological processes within sampling locations is necessary (Matson et al., 2000). Ideally, samples collected prior to the Chernobyl accident and those exposed to the highest dose rates in the immediate aftermath would be available for population genetic analysis. However, such samples were not available. Future research into the effects of chronic radiation exposure on genetic variation should aim to couple sampling over long time scales with a robust understanding of community dynamics.

To the authors' knowledge, this study is the first to apply a genome‐wide SNP approach to studying genetic diversity in response to radiation exposure. The majority of available studies have used techniques such as AFLP, RFLP, or microsatellites, though recent studies have moved toward deep sequencing (e.g., Baker et al., 2017). Given the advantages of genome‐wide SNP discovery techniques as compared to previous approaches (Schlötterer, 2004), this method would have increased analytic power to detect a relationship between radiation dose rate and genetic diversity. The sequencing depth in the present study (>20x) is known to provide accurate genotype calling and de novo SNP discovery even in nonmodel organisms without a reference genome (Andrews, Good, Miller, Luikart, & Hohenlohe, 2016; Torkamaneh et al., 2016). Furthermore, the number of SNPs studied in the present study (14,463 following data filtration) has been shown to provide reliable measures of genetic diversity in nonmodel organisms. For example, Hoffman et al. (2014) found that inbreeding coefficients generated from RAD‐seq analysis of 13,000 SNPS in the oldfield mouse, *Peromyscus polionotus subgriseus*, had strong concordance with known pedigree based values.

## 5 CONCLUSION

This study represents the first assessment of radiation effects on genetic variation in crustaceans, internationally important model organisms in radioecology (ICRP, 2007). No effect of radiation dose rate on genetic variation was recorded, which is different to the majority of available literature recording increased genetic diversity and elevated mutation rates in nonhuman organisms at Chernobyl. This was largely attributed to lower overall dose rates over time as compared to more radiosensitive organisms such as pines and rodents wherein clear genetic effects have been demonstrated. Coupled with previous studies demonstrating no effects of radiation on reproduction and development (Fuller et al., 2018, 2017) in crustaceans at Chernobyl, this study will aid in understanding the long‐term effects of radiation exposure on populations of aquatic biota.

## CONFLICT OF INTEREST

None declared.

## AUTHOR CONTRIBUTIONS

A.T.F, J.T.S, and N.F.: conceived and designed the study. N.F., D.I.G., A.L., and L.L.N.: conducted field sampling. N.F.: performed the laboratory work (DNA extractions and quality control), and N.F., J.T.S, and A.T.F.: wrote the manuscript.

## Data Availability

Filtered SNP, genetic distance (*F*_st_), and heterozygosity data are available through the Open Science Framework (https://doi.org/10.17605/OSF.IO/7WH5A). Other genetic diversity data are available through the Natural Environment Research Council (NERC) Environmental Information Data Centre (EIDC, https://doi.org/10.5285/47f036c4-e319-4825-9cb8-f27977eb20dd).
